# Impact of cleaning and disinfection procedures on microbial ecology and *Salmonella* antimicrobial resistance in a pig slaughterhouse

**DOI:** 10.1038/s41598-019-49464-8

**Published:** 2019-09-10

**Authors:** Arnaud Bridier, Patricia Le Grandois, Marie-Hélène Moreau, Charleyne Prénom, Alain Le Roux, Carole Feurer, Christophe Soumet

**Affiliations:** 1 Antibiotics, Biocides, Residues and Resistance Unit, Fougères Laboratory, ANSES, Fougères, France; 2 Chlean Pass Joint Technological Network, Hygienic Design of Production Lines and Equipment, France; 3 Department of Fresh and Processed Meat, IFIP-Institut du Porc, Maisons-Alfort, France; 4 Department of Fresh and Processed Meat, IFIP-Institut du Porc, Le Rheu, France

**Keywords:** Microbiology, Antimicrobial resistance, Microbial ecology

## Abstract

To guarantee food safety, a better deciphering of ecology and adaptation strategies of bacterial pathogens such as *Salmonella* in food environments is crucial. The role of food processing conditions such as cleaning and disinfection procedures on antimicrobial resistance emergence should especially be investigated. In this work, the prevalence and antimicrobial resistance of *Salmonella* and the microbial ecology of associated surfaces communities were investigated in a pig slaughterhouse before and after cleaning and disinfection procedures. *Salmonella* were detected in 67% of samples and isolates characterization revealed the presence of 15 PFGE-patterns belonging to five serotypes: S.4,5,12:i:-, Rissen, Typhimurium, Infantis and Derby. Resistance to ampicillin, sulfamethoxazole, tetracycline and/or chloramphenicol was detected depending on serotypes. 16S rRNA-based bacterial diversity analyses showed that *Salmonella* surface associated communities were highly dominated by the *Moraxellaceae* family with a clear site-specific composition suggesting a persistent colonization of the pig slaughterhouse. Cleaning and disinfection procedures did not lead to a modification of *Salmonella* susceptibility to antimicrobials in this short-term study but they tended to significantly reduce bacterial diversity and favored some genera such as *Rothia* and *Psychrobacter*. Such data participate to the construction of a comprehensive view of *Salmonella* ecology and antimicrobial resistance emergence in food environments in relation with cleaning and disinfection procedures.

## Introduction

Salmonellosis remains one of the main foodborne zoonosis in Europe with 94.530 cases in 2016^1^. In France, between 2008 and 2013, *Salmonella* was the leading cause of death related to contaminated food (26.2%)^2^. Pig products constitute common sources of human infections involving *Salmonella* and slaughtering line represent a critical stage for controlling its dissemination on the food chain^3,4^. The continuous introduction of bacteria, potentially including *Salmonella*, with each new pig band and the presence of a resident flora associated to the slaughter environment may indeed constitute an important risk for carcass contamination during the slaughtering or meat-cutting processes^5^ despite good hygiene practices. In order to guarantee food safety, a better understanding of *Salmonella* ecology and persistence strategies in such food environment constitutes a prerequisite. In this perspective, the role of interactions with resident flora in pathogenic *Salmonella* survival on the food chain^6^ require a particular interest. Indeed, several studies have showed that formation of biofilm and persistence of *Salmonella* could be promoted by the presence of other bacteria including strains directly isolated from food industries^7,8^. However, data about microbial ecology in pig slaughterhouses are lacking in the literature, thus stressing the need to improve our knowledge on microbial diversity and population dynamics in such food environments.

Alongside, antimicrobial resistance (AMR) in bacteria including foodborne pathogen as *Salmonella* is becoming one of the most preoccupying issues for public health. Being a continuum between environment, animals and human health, food chain constitutes a privileged area for occurrence and spreading of antimicrobial resistance^9,10^. Food processing industries as slaughterhouses for instance can act as connecting paths including substantial selection for resistance due to procedures inherent to the industrial process such as cleaning and disinfection (C&D). In the last few years, an increasing number of studies have indeed highlighted the potential role of biocides in the selection of cross-resistance towards antibiotics in bacteria^11–13^. Nevertheless, the vast majority of them were conducted using pure culture models in laboratory conditions, and the current lack of data makes it difficult to evaluate the substantial risk to select bacteria with higher resistance to antibiotics through the use of biocides in industries and to determine which biocides is associated with the highest risk of antibiotic resistance cross-selection. The need to multiply environmental studies focusing on the characterization of cross-resistance to antibiotics in pathogens following use of biocides and including considerations about complex microbial ecology associated was therefore highlighted^14^.

In this context, this project aimed at collecting data about *Salmonella* prevalence and anti-microbial resistance (AMR) levels in a pig slaughterhouse, gaining an overview of associated bacterial community diversity and to examine how C&D steps impact microbial communities and potentially select isolates with reduced susceptibility to biocides and/or antibiotics.

## Materials and Methods

### Pig slaughterhouse sampling and samples processing

The study was carrying out in a French pig slaughterhouse. The slaughterhouse was visited four times between March and May 2017. Samples were collected from the 6 following areas: 2 were located from the “dirty” zone at the dehairing machine (DH) and whips from the scraping machine (WH), and 4 were taken from the “clean” zone (after a singeing step) on the neck clipper machine (NC), carcass opener circular saw (CO), white offals gutter (WOG) and platform used for red offal removing (ROP). Each area was sampled twice at the same location for each sampling date, before and after C&D procedures. While the cleaning and disinfection procedures are highly dependent from the slaughterhouse, the pig slaughterhouse sampled in this work was chosen because it used biocide formualtions among the most used. Concretely, C&D procedures consisted here in daily application of a chlorinated alkaline solution along chain using foamer after slaughtering following by a rinsing step. An ethanol-based solution was additionally sprayed on cutting blades in contact with meat at NC and CO areas. In addition, an acid foaming solution was applied weekly along the slaughter chain. Samples were collected by swabbing 1 m² of surfaces using sterile wipes and placed individually in sterile Stomacher bags with filters (BagFilter® P400, Interscience, France) which were then transported to the laboratory in refrigerated conditions and processed within 12 h. Upon arrival, bags containing wipes were filled using 225 ml of One broth medium (Oxoid, France) and introduced in a stomacher (BagMixer® 400 P, Interscience, France) for blending during 1 min at high speed. Two aliquots of 20 ml of stomached suspension were directly sampled from bags and centrifuged at 10,000 g during 5 min and pellets were frozen at −80 °C for further DNA extraction and 16s rRNA sequencing. An additional aliquot of 1 ml was used to enumerate total bacterial flora after serial 10-fold dilutions in peptone water and plating on tryptone soya agar (TSAye, Beckton-dickinson, France). Differences in bacterial counts between sampling area or before and after disinfection were analyzed statistically using analysis of variance (ANOVA, significance for *p* < 0.05). One aliquot of 10 ml was also directly frozen at −80 °C for sample conservation.

### Salmonella enrichment, isolation and PCR confirmation

The *Salmonella Precis*^TM^ protocol validated by AFNOR to ISO 16140 standard was used according to manufacturer instructions for the enrichment, detection and confirmation of *Salmonella*. Concretely, after stomaching, bags containing wipes in One broth medium (Oxoid, France) were transferred at 42 °C for enrichment. After 20–24 h, 10 µl were plated on Brilliance Salmonella agar (Oxoid, France) and incubated 24 h at 37 °C before identification of positive *Salmonella* colonies according to manufacturer instructions. *Salmonella* were then re-isolated on TSAye and incubated 20 h at 37 °C before being stocked at −80 °C in cryoprotective solution or used for PCR confirmation. PCR was performed using forward primer ST11 5′-GCCAACCATTGCTAAATTGGCGCA-3′ and reverse primer ST15 5′-GGTAGAAATTCCCAGCGGGTACTGG-3′^15^ and a LightCycler 480 thermocycler (Roche, France) as follows: 95 °C for 5 min for initial melting; 40 cycles at 95 °C for 30 s, 59 °C for 30 s, 72 °C for 30 s; and 72 °C for 10 min following by incubation at 4 °C.

### Serotyping

*S*erotyping was performed according to the White-Kauffmann-Le Minor scheme (refs. ^16,17^).

### Pulse field gel electrophoresis (PFGE)

PFGE using XbaI restriction enzyme was carried out with a CHEF-DR III system (Bio-Rad), according to the PulseNet Protocol^18^. *Salmonella enterica* serotype Braenderup H9812 was used as the molecular size marker in the PFGE experiment^19^. Gels were stained with ethidium bromide and banding patterns were visualized under UV light, using the Gel Doc XR and Quantity One software (Bio-Rad). DNA patterns were analyzed with BioNumerics software (V 7.6.3, Applied Maths, Kortrijk, Belgium). Algorithms available within the program were used to compare patterns. Dendrograms were produced, using the Dice coefficient and the unweighted pair group method with arithmetic averages (UPGMA), with a 1% tolerance limit and 1% optimization^20^. Each PFGE pattern differing by at least one band from a previously recognized type was considered to be a new pattern. Each new pattern was given a unique designation, as suggested by Peters *et al*.^21^, and added to the PFGE patterns library. The recommendations of Barrett *et al*.^22^ were also followed for the interpretation of PFGE patterns.

### Biocide susceptibility testing

Minimal inhibitory concentrations (MIC) were determined using a standard microdilution method for the 3 biocide solutions used in the slaughterhouse: a chlorinated alkaline solution, an ethanol-based solution and an acid foaming solution. Bacterial inocula were prepared from overnight cultures diluted in Muller-Hinton broth to reach from 1 to 3.10^5^ CFU/well in presence of various concentrations of biocides in a 96-wells microtiter plate (Greiner Bio-one 650101, France). Plates were then incubated at 37 °C for 24 h and MIC was determined as the lowest concentration of biocide that prevents bacterial growth. All determinations of MIC were repeated twice. Statistical differences between MIC distribution for biocides between strains isolated before and after C&D procedures or between serotypes were assessed using a Student test (significance for *p* < 0.05).

### Antibiotic susceptibility testing

Antibiotic susceptibility tests were performed using a standard microdilution method with the Sensititre® system on EUVSEC plates (Trek Diagnostic Systems, UK) using a panel of 14 antimicrobial substances according to manufacturer instructions. The strains were interpreted as resistant to antibiotics according to the epidemiological resistance cut-off determined from EUCAST (European Committee on Antimicrobial Susceptibility Testing, http://mic.eucast.org).

### DNA extraction, PCR amplification and 16s rRNA sequencing

Frozen pellet obtained from 20 ml sample extract after stomaching were centrifuged and DNA extraction was performed on the pellet. Nucleospin® tissue kit (Macherey-Nagel) was used for the extraction according to manufacturer instructions. Concentration and quality of extracted DNA were checked using a BioSpec-nano spectrophotometer (Shimadzu). The bacterial V3-V4 region of the 16S rRNA gene was then amplified using the forward primer 5′-CTTTCCCTACACGACGCTCTTCCGATCTACGGRAGGCAGCAG-3′ and the reverse primer 5′-GGAGTTCAGACGTGTGCTCTTCCGATCTTACCAGGGTATCTAATCCT-3′^23^. The preparation of amplicons was performed in a total volume of 50 µL containing 0.5 µl of TAQ Polymerase (5 U/µl) and 5 µl of adequate 10 X PCR buffer (MTP Taq DNA Polymerase, Sigma), 1 µl of 10 mM dNTP (Sigma), 1.25 µM of each primer (20 µM) and 10 ng of DNA template. PCR was performed using a LightCycler 480 thermocycler as follows: 94 °C for 60 s for initial melting; 30 cycles at 94°c for 60 s, 65 °C for 60 s, 72 °C for 60 s; and 72 °C for 10 min following by incubation at 4 °C. Products were then verified on a 1.5% agarose gel before being sent to GeT-PLaGe Genotoul plateform (Castanet-Tolosan, France) for paired-end sequencing on Illumina MiSeq platform (Illumina, USA) at a read length of 2 × 250 pb.

### Bioinformatics analyses

Illumina sequences were processed using the FROGS pipeline (Find Rapidly OTU with Galaxy Solution)^24^ implemented on a galaxy instance (http://migale.jouy.inra.fr/galaxy/). Bacterial 16S rRNA paired-end reads were merged with a maximum of 10% mismatches in the overlap region using FLASH^25^. Denoising procedures consisted in discarding reads with no expected length and the ones containing ambiguous bases (N). After dereplication, the clusterisation tool ran with SWARM^26^ with an aggregation distance equal to 3. Chimeras were then removed using VSEARCH^27^ and sequences were filtered to keep OTUs (also called *clusters*) accounting for at least 0.005% of all sequences^28^. Taxonomic affiliation was performed with both RDP Classifier^29^ and Blastn+^30^ against the SILVA 132 pintail 100 database^31^.

Data set was rarefied and alpha diversity indexes (observed OTU, Chao1, Shannon and InvSimpson) were calculated and further plotted using the R “phyloseq” package^32^. Principal coordinate analysis (PCoA) were performed on weighted unifrac dissimilarity matrices calculated using the R package phyloseq. Using non-parametric permutation-based multivariate analysis of variance (PerMANOVA, function adonis in R package “vegan”) on unifrac and weighted unifrac-based distance matrices, we tested for significant differences in community structure. Assumptions of the adonis test were verified using the betadisper function in the R package vegan, which tests the multivariate homogeneity of group dispersions. Pairwise comparisons were performed on weighted unifrac distance matrix using pairwise.adonis function from “vegan” package and Bonferroni *p*-value correction (significance for *p* < 0.05). Figures were prepared with R and the package ggplot2.

Differentially abundant taxa in bacterial populations depending on various factors were identified using the linear discriminant analysis (LDA) effect size (LEfSe)^33^. Relative abundances of all features were first compared by using the nonparametric Kruskal-Wallis rank sum test (significance for *p* < 0.05), and each statistically significant feature was further subjected to effect size estimation using LDA.

## Results

### Sampling, isolation and typing of *Salmonella*

A total of 48 wipes taken before (24) and after (24) disinfection at six different sites in a pig slaughterhouse and at four different dates were collected. Total flora enumeration showed similar bacterial counts for the different areas sampled with values comprised between 3.34 ± 1,87 and 4.68 ± 0.56 log (CFU)/cm² except for the dehairing area (DH) before C&D procedures which exhibited significant higher bacterial counts with 6.15 ± 0.26 log (CFU)/cm² (see Table 1). There was no significant difference between bacterial counts between samples taken before and after C&D, except for the dehairing area with a significant decrease (*p* < 0.05) from 6.15 ± 0.26 to 4.66 ± 0.79 log (CFU)/cm² after C&D.

**Table 1:** Distribution of *Salmonella* isolates in the pig slaughterhouse.

Date	DH		WH		NC		CO		WOG		ROP	
	Bef.	Aft.	Bef.	Aft.	Bef.	Aft.	Bef.	Aft.	Bef.	Aft.	Bef.	Aft.
03/22/2017	B05	B05	B06						***B13***	B06	B01, B15	*B11*, B06
04/03/2017	B01	B01	***B08***, ***B14***	B09, B16			B12	B12	*B10*	B01, *B11*		*B10*
04/24/2017	**B09**	**B09**						B12	**B09**	**B09**	**B09**	
05/15/2017	B04	B04	B01	B03, B01			B12	B04	B04	B02	B01	B01
Tot. bact. count (Log(CFU/cm^2^)	6.15 ± 0,26	4.66 ± 0,79	3.79 ± 0,73	4.13 ± 0,77	3.46 ± 0,51	4.10 ± 1,57	3.34 ± 1,87	3.56 ±0,66	3.59 ± 1,07	4.68 ± 0,56	3.77 ± 1,26	3.91 ± 0,61

The table shows the presence of different PFGE patterns at the different sampling area and date, each time before (Bef.) and after (Aft.) C&D for isolates of S. monophasic 4,5,12:i:- (Black), Typhimurium (Bold), Rissen (Italic), Infantis (Underline) and Derby (Bold/Italic). The mean total bacterial counts (CFU/cm²) at the different sampling areas in the slaughterhouse are also indicated. DH: dehairing, WH: whipping, NC: neck-clipper, CO: carcass opener, WOG: white offal gutter, ROP: red offal platform.

A total of 38 *Salmonella* strains were isolated from 32 samples (67% of positive samples) with 18 and 20 strains isolated before and after C&D procedures respectively. There was no significant difference between numbers of positive samples before and after C&D procedures. All sites investigated were positive for *Salmonella* except the neck clipper for which *Salmonella* was never isolated (Table 1). PFGE and serotyping analyses revealed 15 PFGE patterns belonging to the following 5 serotypes: S. 4,5,12:i:-, Typhimurium, Rissen, Infantis and Derby. The monophasic Typhimurium variant 4,5,12:i:- represented 58% of strains isolated and showed 9 distinct PFGE patterns (B01, B02, B03, B04, B05, B06, B09, B15 and B16), serotype Typhimurium represented 13% of isolates and showed 1 PFGE pattern (B09), serotype Rissen accounted for 10.5% of isolated *Salmonella* and presented 2 PFGE patterns (B10 and B11), serotype Infantis reached 10.5% of the total number of isolates with a unique PFGE patterns (B12) and serotype Derby represented 8% of strains isolated and was subdivided in 3 PFGE patterns (B08, B13 and B14). As showed in Table 1, while monophasic Typhimurium variant serotype 4,5,12:i:- was found at all *Salmonella*-positive sampling area and for various dates, serotype Typhimurium was only isolated at a single date (04/24) at DH, WOG and ROP areas. Interestingly, serotype Infantis was exclusively found at CO area but repeatedly for three of the four dates investigated. Serotypes Rissen and Derby were occasionally isolated both at two different dates (03/22 and 04/03) and sampling areas (WH and WOG for Derby and WOG and ROP areas for Rissen).

### Antibiotic resistance profiles

Antibiotic susceptibilities of the 38 isolated *Salmonella* strains were obtained for a total of 14 antibiotics. MIC values obtained are available in Table 2. Resistances to ampicillin, sulfamethoxazole and tetracycline were observed for all PFGE patterns of the monophasic Typhimurium variant 4,5,12:i:- serotype except for the PFGE pattern B02 which was susceptible to all antibiotics tested. Two of the three PFGE patterns of the Derby serotype exhibited resistance to sulfamethoxazole and tetracycline, the third being susceptible to the 14 antibiotics (type B13). Strains of serotype Rissen were all resistant to tetracycline. Strains of serotype Typhimurium were resistant to ampicillin, chloramphenicol, sulfamethoxazole and tetracycline. Strains from serotype Infantis were susceptible to all tested antibiotics. There was no marked increase in number of resistant strains comparing isolates before and after C&D procedures for the different antibiotics tested. 66.7 and 70.6% of isolated strains were resistant to ampicillin, 16.7 and 11.8% to chloramphenicol, 77.8 and 70.6 to sulfamethoxazole, 83.3 and 82.4 to tetracycline before and after C&D, respectively. In addition, none of the persistent PFGE patterns which were isolated repeatedly at a given area both before and after C&D showed a significant change in MIC values of more than one dilution for the 14 antibiotics tested (Table 2).

**Table 2:** Minimal inhibitory concentrations of 14 antibiotics for the 38 *Salmonella* isolates.

Source		Date	Serotype	PFGE pattern	Antibiotics (mg/l)													
					AMP	AZI	FOT	TAZ	CHL	CIP	COL	GEN	MER	NAL	SMX	TET	TIG	TMP
ROP	Bef.	03/22	4,5,12:i:-	B01	**>64**	4	≤0,25	≤≤0,5	≤8	0,03	≤1	≤0,5	≤0,03	≤4	**>1024**	**>64**	0,5	≤0,25
DH	Bef.	04/03		B01	**>64**	4	≤0,25	≤0,5	≤8	0,03	≤1	≤0,5	≤0,03	≤4	**>1024**	**>64**	0,5	≤0,25
DH	Aft.	04/03		B01	**>64**	8	≤0,25	≤0,5	≤8	0,03	≤1	≤0,5	≤0,03	≤4	**>1024**	**>64**	≤0,25	≤0,25
WOG	Aft.	04/03		B01	**>64**	8	≤0,25	≤0,5	≤8	0,03	≤1	≤0,5	≤0,03	≤4	**>1024**	**>64**	0,5	≤0,25
ROP	Bef.	05/15		B01	**>64**	4	≤0,25	≤0,5	≤8	0,03	≤1	≤0,5	≤0,03	≤4	**>1024**	**>64**	0,5	≤0,25
ROP	Aft.	05/15		B01	**>64**	8	≤0,25	≤0,5	≤8	0,03	≤1	≤0,5	≤0,03	≤4	**>1024**	**>64**	0,5	≤0,25
WH	Bef.	05/15		B01	**>64**	4	≤0,25	≤0,5	≤8	0,03	≤1	≤0,5	≤0,03	8	**>1024**	**>64**	0,5	≤0,25
WH	Aft.	05/15		B01	**>64**	4	≤0,25	≤0,5	≤8	0,03	≤1	≤0,5	≤0,03	8	**>1024**	**>64**	0,5	≤0,25
WOG	Aft.	05/15		B02	2	8	≤0,25	≤0,5	≤8	0,03	≤1	≤0,5	≤0,03	≤4	32	≤2	0,5	≤0,25
WH	Aft.	05/15		B03	**>64**	4	≤0,25	≤0,5	≤8	0,03	≤1	≤0,5	≤0,03	≤4	**>1024**	**>64**	0,5	≤0,25
CO	Aft.	05/15		B04	**>64**	4	≤0,25	≤0,5	≤8	0,03	≤1	≤0,5	≤0,03	≤4	**>1024**	**>64**	0,5	≤0,25
DH	Bef.	05/15		B04	**>64**	8	≤0,25	≤0,5	≤8	0,03	≤1	≤0,5	≤0,03	≤4	**>1024**	**>64**	≤0,25	≤0,25
DH	Aft.	05/15		B04	**>64**	8	≤0,25	≤0,5	≤8	0,03	≤1	≤0,5	≤0,03	≤4	**>1024**	**>64**	0,5	≤0,25
WOG	Bef.	05/15		B04	**>64**	8	≤0,25	≤0,5	≤8	0,03	≤1	≤0,5	≤0,03	≤4	**>1024**	**>64**	0,5	≤0,25
DH	Bef.	03/22		B05	**>64**	8	≤0,25	≤0,5	≤8	0,03	≤1	≤0,5	≤0,03	≤4	**>1024**	**>64**	0,5	≤0,25
DH	Aft.	03/22		B05	**>64**	8	≤0,25	≤0,5	≤8	0,03	≤1	≤0,5	≤0,03	≤4	**>1024**	**>64**	≤0,25	≤0,25
ROP	Aft.	03/22		B06	**>64**	8	≤0,25	≤0,5	≤8	0,03	≤1	≤0,5	≤0,03	≤4	**>1024**	**>64**	≤0,25	≤0,25
WH	Bef.	03/22		B06	**>64**	8	≤0,25	≤0,5	≤8	0,03	≤1	≤0,5	≤0,03	≤4	**>1024**	**>64**	0,5	≤0,25
WOG	Aft.	03/22		B06	**>64**	8	≤0,25	≤0,5	≤8	0,03	≤1	≤0,5	≤0,03	≤4	**>1024**	**>64**	≤0,25	≤0,25
ROP	Bef.	03/22		B15	**>64**	8	≤0,25	≤0,5	≤8	0,03	≤1	≤0,5	≤0,03	≤4	**>1024**	**>64**	≤0,25	≤0,25
WH	Aft.	04/03		B16	**>64**	8	≤0,25	≤0,5	≤8	0,03	≤1	≤0,5	≤0,03	≤4	**>1024**	**>64**	≤0,25	≤0,25
WH	Aft.	04/03		B09	**>64**	8	≤0,25	≤0,5	≤8	0,03	≤1	≤0,5	≤0,03	≤4	**>1024**	**>64**	0,5	≤0,25
WH	Bef.	04/03	Derby	B08	2	8	≤0,25	≤0,5	≤8	0,03	≤1	≤0,5	0,06	≤4	**>1024**	**>64**	0,5	0,5
WOG	Bef.	03/22		B13	2	8	≤0,25	≤0,5	≤8	≤0,015	≤1	≤0,5	0,06	≤4	32	≤2	0,5	≤0,25
WH	Bef.	04/03		B14	2	8	≤0,25	≤0,5	≤8	0,03	≤1	≤0,5	0,06	≤4	**>1024**	**>64**	0,5	0,5
CO	Bef.	04/03	Infantis	B12	2	8	≤0,25	≤0,5	≤8	0,03	≤1	≤0,5	≤0,03	≤4	32	≤2	0,5	≤0,25
CO	Aft.	04/03		B12	2	8	≤0,25	≤0,5	≤8	0,03	≤1	≤0,5	≤0,03	≤4	32	≤2	≤0,25	0,5
CO	Aft.	24/04		B12	2	8	≤0,25	≤0,5	≤8	0,03	≤1	≤0,5	≤0,03	≤4	32	≤2	0,5	≤0,25
CO	Bef.	05/15		B12	2	8	≤0,25	1	≤8	0,03	≤1	≤0,5	0,06	≤4	32	≤2	0,5	0,5
ROP	Aft.	04/03	Rissen	B10	2	16	≤0,25	≤0,5	≤8	0,03	≤1	≤0,5	≤0,03	≤4	32	**>64**	0,5	0,5
WOG	Bef.	04/03		B10	2	8	≤0,25	1	≤8	0,03	≤1	2	≤0,03	≤4	32	**>64**	0,5	0,5
ROP	Aft.	03/22		B11	2	8	≤0,25	≤0,5	≤8	≤0,015	≤1	≤0,5	≤0,03	≤4	32	**>64**	0,5	0,5
WOG	Aft.	04/03		B11	2	8	≤0,25	≤0,5	≤8	≤0,015	≤1	≤0,5	≤0,03	≤4	32	**>64**	0,5	0,5
DH	Bef.	24/04	Typhimurium	B09	**>64**	4	≤0,25	≤0,5	**>128**	0,03	≤1	≤0,5	0,06	≤4	**>1024**	**32**	≤0,25	0,5
DH	Aft.	24/04		B09	**>64**	4	≤0,25	≤0,5	**>128**	0,03	≤1	≤0,5	≤0,03	≤4	**>1024**	**32**	≤0,25	≤0,25
ROP	Bef.	24/04		B09	**>64**	4	≤0,25	≤0,5	**>128**	0,03	≤1	≤0,5	≤0,03	≤4	**>1024**	**32**	≤0,25	≤0,25
WOG	Bef.	24/04		B09	**>64**	8	≤0,25	≤0,5	**>128**	0,03	≤1	≤0,5	0,06	≤4	**>1024**	**32**	≤0,25	≤0,25
WOG	Aft.	24/04		B09	**>64**	8	≤0,25	≤0,5	**>128**	0,03	≤1	≤0,5	≤0,03	≤4	**>1024**	**32**	≤0,25	0,5

Strains were isolated from the different areas (Bef.) before or (Aft.) after C&D procedures. Values in bold indicate resistance according to the epidemiological cut-off determined from EUCAST. DH: dehairing, WH: whipping, NC: neck-clipper, CO: carcass opener, WOG: white offal gutter, ROP: red offal platform. AMP: ampicillin, AZI: azithromycin, FOT: cefotaxim, CHL: chloramphenicol, CIP: ciprofloxacin, COL: colistin, GEN: gentamicin, MER: meropenem, NAL: nalidixic acid, SMX: sulfamethoxazol, TAZ: ceftazidin, TET: tetracycline, TGC: tigecyclin, TMP: trimethoprim.

### Susceptibility of *Salmonella* isolates to biocides

**Figure 1:**
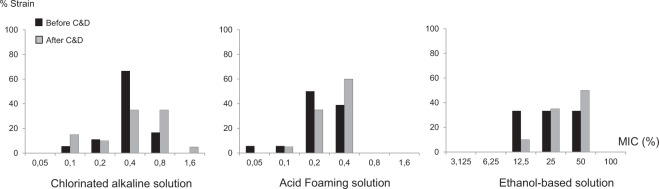
Minimal inhibitory concentrations (MIC) for the three biocides used in the slaughterhouse. Distribution of MIC values for the chlorinated alkaline solution, acid solution and ethanol base solution are compared before and after C&D.

MICs of the three biocide solutions used in the slaughterhouse were determined using a broth microdilution method. MIC distributions for the *Salmonella* isolated before and after C&D procedures are displayed in Fig. 1 for the three biocides used in the slaughterhouse. MIC values were comprised between 0.1 and 1.6% for chlorinated alkaline solution, 0.05 and 0.4% for acid solution and 12.5 and 50% for ethanol-based solution. There was no significant difference (p > 0.05) when comparing MIC distributions between strains isolated before and after C&D for these three biocides. C&D procedures used here thus did not lead to the selection of strains with lower susceptibility toward biocides during the two months of observation. There was also no significant difference between the serotypes for the different biocide tested (p > 0.05).

### Microbial surface community diversity

A total of 47 samples were sequenced successfully (the sample from the 03/22 before C&D at the carcass opener area (CO) did not yield sufficient DNA for sequencing). After filtering for quality, length and removal of chimera, a total of 1 175,969 sequences were obtained corresponding to an average read number per sample of 25,020 ± 11,073 reads. A total of 441 operational taxonomic units (OTUs) were obtained after bioinformatics analysis.

**Figure 2:**
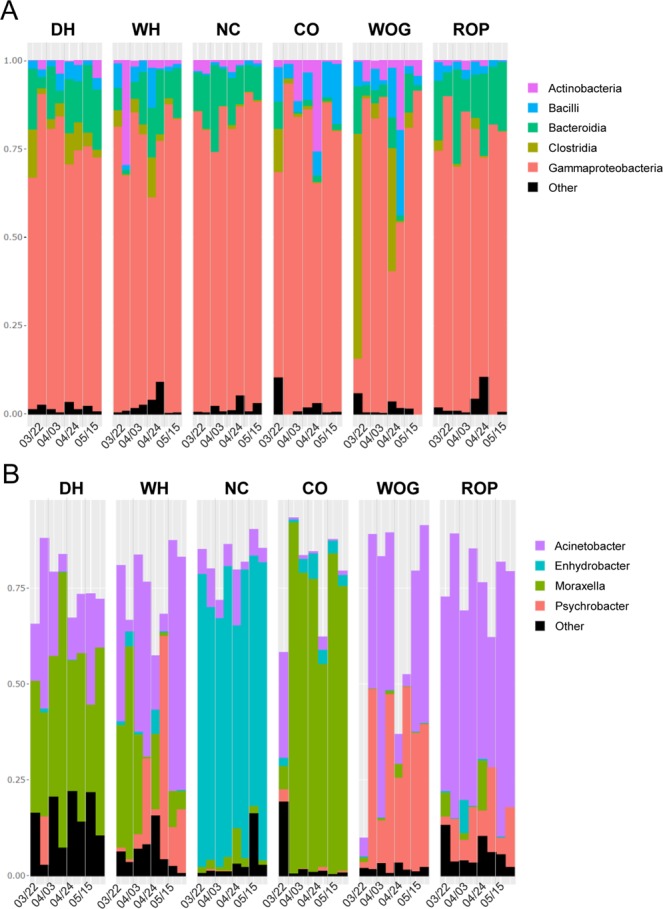
Taxonomic composition of bacterial communities at the different sampling area in the slaughterhouse. For each sampling area, two bars for each of the 4 sampling dates are presented and correspond to communities before and after C&D. (**A**) Each bar represents relative abundances of the 5 top bacterial classes in samples. (**B**) Bar plots display the relative abundance of the four most abundant genera in *gamma-proteobacteria*. DH: dehairing, WH: whipping, NC: neck-clipper, CO: carcass opener, WOG: white offal gutter, ROP: red offal platform.

Taxonomic composition of samples revealed a high dominance of *gamma-proteobacteria* in the slaughterhouse with an average relative abundance of 75.8 ± 5.3% (Fig. 2A). Following dominant bacterial classes identified were *Bacteroidia*, *Clostridia*, *Bacilli* and *Actinobacteria*. The four genera*Enhydrobacter*, *Moraxella*, *Acinetobacter* and *Psychrobacter*, all belonging to the *Moraxellaceae* family, clearly dominated bacterial populations with a specific composition depending on sampling area as showed in Fig. 2B. The dehairing area was dominated by *Moraxella* and *Acinetobacter* genus. The whipping area exhibited similar bacterial composition but with high amounts of *Psychrobacter* for some samples. The genus *Enhydrobacter* clearly dominated the neck clipper area with relative abundance comprised between 52.8 and 77.7%. The carcass opener area was overall dominated by the *Moraxella* Genus which reached up to 91.7% of relative abundance but with one sample showing a specific composition with 6.1% of *Moraxella* and 27.5% of *Acinetobacter*. *Acinetobacter* and *Psychrobacter* dominated white offal gutter and red offal platform areas but with a higher relative abundance of *Acinetobacter* at red offal platform with relative abundances from 33.9 to 74.3%. In addition, analysis of bacterial population structures at the OTU (cluster) level (Fig. S1) revealed that *Enhydrobacter*, *Moraxella* or *Psychrobacter* populations were each dominated by only 1 or 2 recurrent OTUs for all the sampling areas. *Acinetobacter* genus demonstrated a higher diversity including a total of 34 different OTUs and a higher variation of OTUs proportions depending on sampling area.

**Figure 3:**
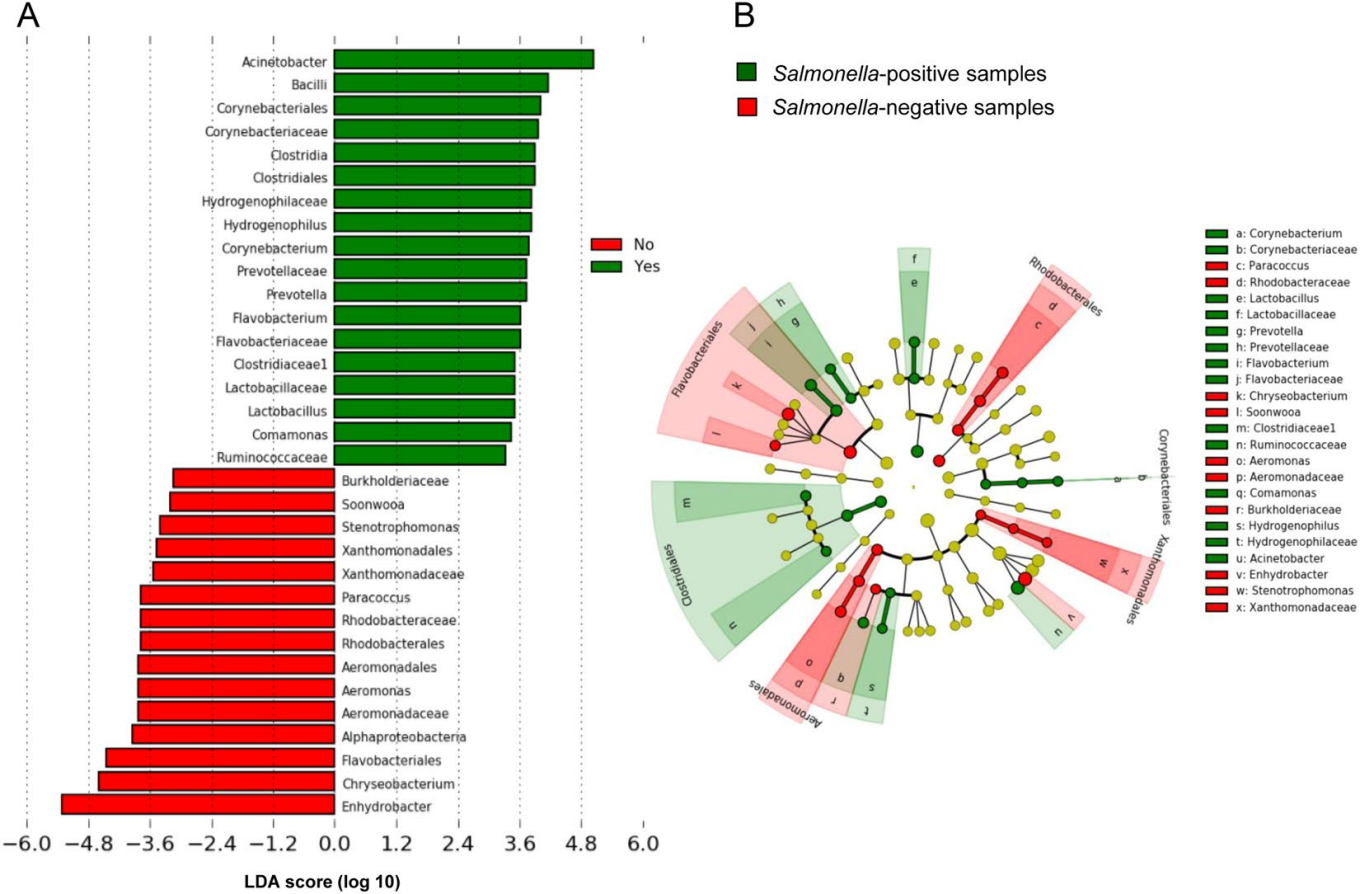
Bacterial taxa significantly differentiated between *Salmonella*-positive and *Salmonella*-negative populations identified by linear discriminant analysis coupled with effect size (LefSE). (**A**) LDA score obtained for differentially abundant taxa. Only taxa with a significant LDA threshold value of >3 are displayed. (**B**) Circular cladogram reporting LEfSe results presenting the identified taxa distributed according to phylogenetic characteristics.

LEfSE analysis was then used to identify differentially abundant bacterial taxa between *Salmonella*-positive and *Salmonella*-negative populations (Fig. 3). *Flavobacteriales*, *Rhodobacterales*, *Xanthomonadales* and *Aeromonadales* orders were significantly more abundant in *Salmonella*-negative samples whereas *Clostridiales* and *Corynebacteriales* orders were more abundant in *Salmonella*-positive communities. Six genera including *Enhydrobacter*, *Chryseobacterium*, *Aeromonas*, *Paracoccus*, *Stenotrophomonas* and *Soonwooa* (from higher to lower LDA scores) were significantly more abundant in *Salmonella*-negative populations. Conversely, *Acinetobacter*, *Hydrogenophilus*, *Corynebacterium*, *Prevotella*, *Flavobacterium*, *Lactobacillus*, and *Comamonas* (from higher to lower LDA scores) were significantly more abundant in samples harboring *Salmonella*.

**Figure 4:**
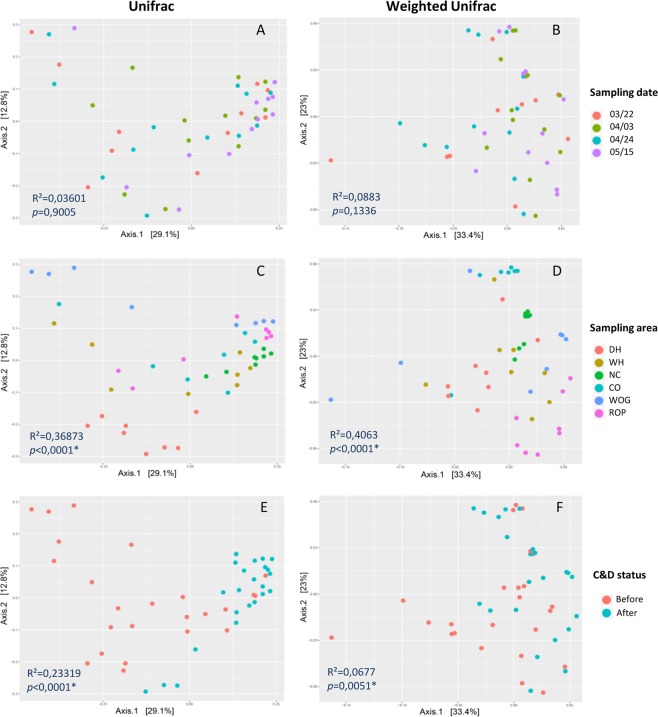
Principal coordinate analysis (PCoA) based on Unifrac and weighted Unifrac distances between samples from slaughterhouse surfaces. Samples were displayed by (**A**,**B**) sampling dates, (**C**,**D**) by sampling areas and (**E**,**F**) depending on C&D status. Adonis (PERMANOVA) statistics are also indicated. First two components explained about 41.9% and 56.4% of total variance in the dataset for Unifrac and weighted Unifrac distance matrices respectively. DH: dehairing, WH: whipping, NC: neck-clipper, CO: carcass opener, WOG: white offal gutter, ROP: red offal platform.

A principal coordinate analysis (PCoA) using Unifrac and weighted Unifrac distances was performed to visualize differences in microbial population structures among samples depending on sampling areas, sampling dates or between samples before and after C&D (Fig. 4). While PERMANOVA did not show significant difference in microbial population structures among samples between sampling dates (*p* = 0.9905 and 0.1336 for unifrac and weighted unifrac distance respectively, Fig. 4A,B, significant effects were observed with regards to sampling area (*p* < 0.0001, Fig. 4C,D). Pairwise comparisons using Adonis (confirmed significant difference (*p* < 0.05) between all sampling areas except between WH and WOG, ROP, DH for unifrac distance and between WH andWOG, ROP, DH and CO for weighted unifrac distance, stressing the heterogeneity of WH populations depending on sampling date compared to the other sampling areas. The homogeneity of dispersion between each sampling area was checked using the betadisper function and revealed that NC area demonstrated significantly (*p* < 0.05) a different dispersion compared with CO and WOG for unifrac distance and only with WOG for weighted unifrac distance.

**Figure 5:**
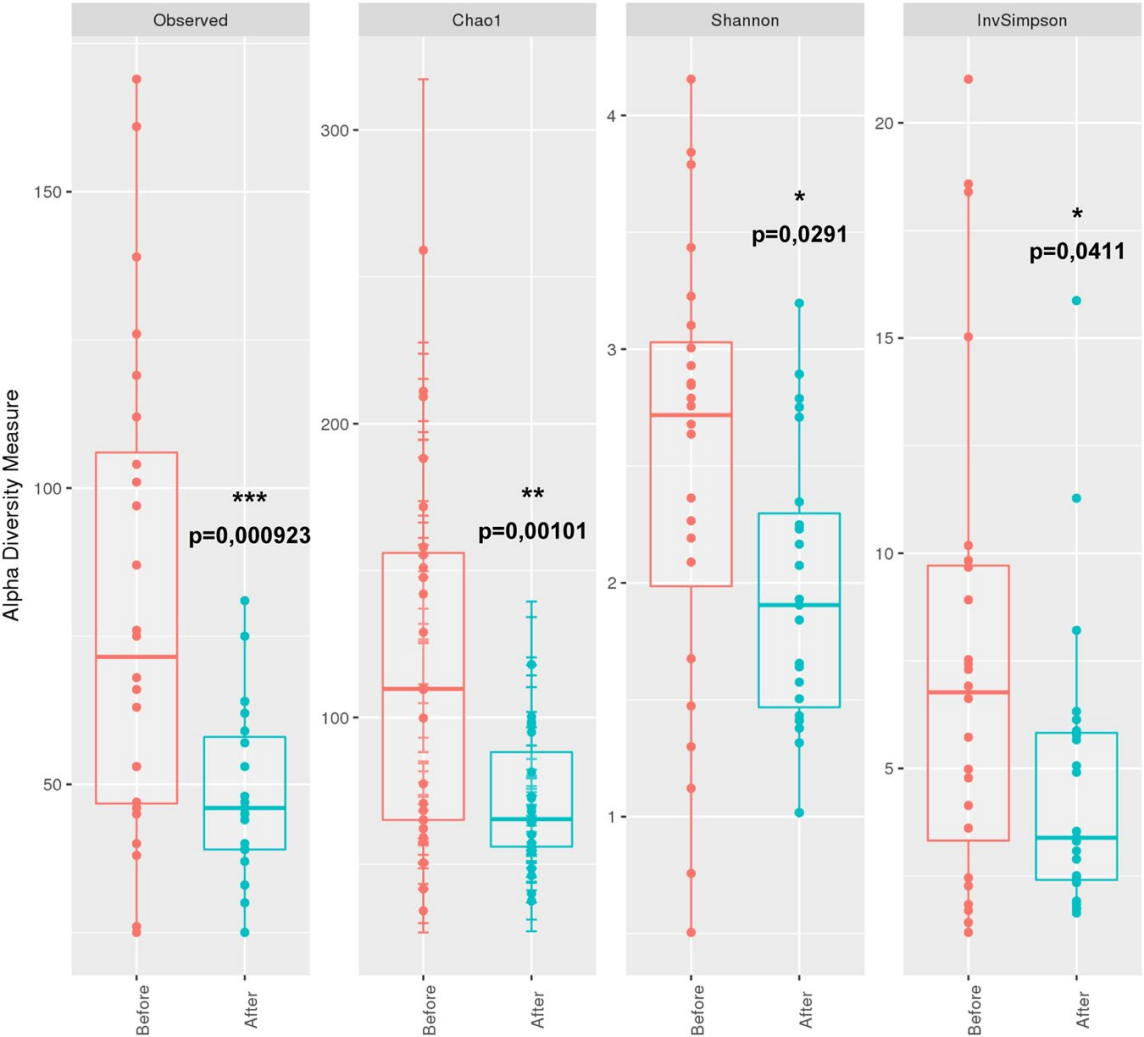
Comparison of α-diversity indexes of microbial populations before and after C&D procedures.

**Figure 6:**
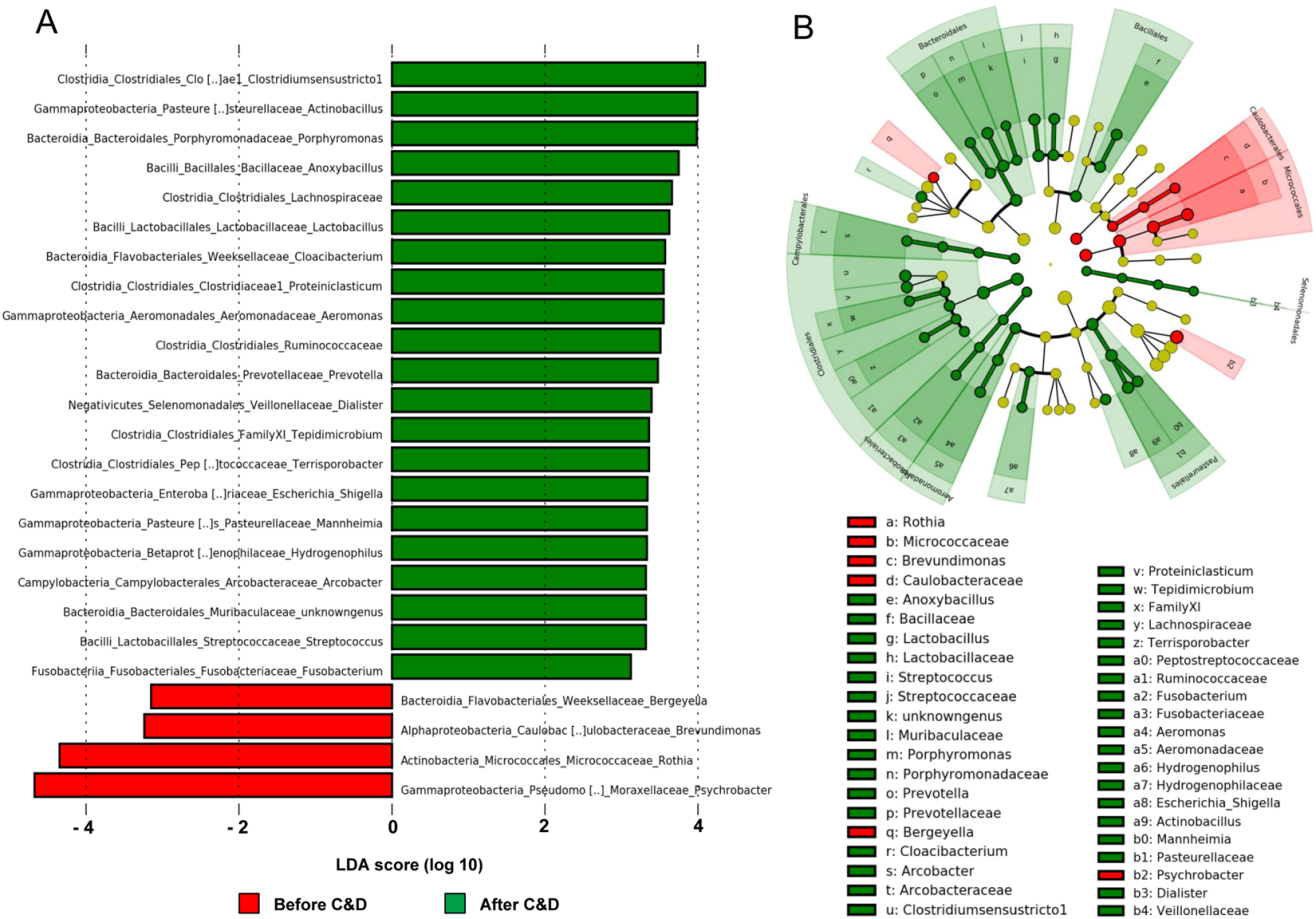
Bacterial genera significantly differentiated between populations sampled before or after C&D identified by linear discriminant analysis coupled with effect size (LefSE). (**A**) LDA score obtained for differentially abundant genus between sample before and after C&D. Only taxa with a significant LDA threshold value of >3 are displayed. (**B**) Circular cladogram reporting LEfSe results presenting the identified taxa distributed according to phylogenetic characteristics.

A significant difference was also observed between composition of bacterial populations sampled before or after C&D procedures for both unifrac (*p* < 0.0001 *and R*² = 0.23319) and weighted unifrac (*p* = 0.0051 and, *R*² = 0.0677) distances (Fig. 4). Unifrac distance PCoA (Fig. 4E) led to a better clustering of samples depending of C&D status in comparison to weighted unifrac PCoA (Fig. 4F). The comparison of α-diversity indexes (number of observed and estimated (Chao1) OTUs, Shannon and inverse Simpson indexes) showed a significant decrease for all the α-diversity index values corresponding to a decrease of richness and evenness in bacterial populations after C&D procedures (Fig. 5). To identify bacterial taxa with the greatest difference in abundance between populations before or after C&D, a LEfSE analysis using OTUs with relative abundance >1% was performed (Fig. 6). Bacterial genera more abundantly represented before (green) or after (red) C&D with LDA score and associated cladogram were displayed in Fig. 6. Numerous orders including *Aeromonadales*, *Fusobacteriales*, *Clostridiales*, *Campylobacterales*, *Bacteroidales*, *Bacillales*, *Selenomonadales and Pasteurellales* were more abundant in samples taken before C&D. Conversely, genera *Bergeyella*, *Brevundimonas* (and *Caulobacterales* order), *Rothia* (and *Micrococcales* order) and *Psychrobacter* were significantly more abundant (*p* < 0.05) in samples after C&D procedures with LDA score >3. Interestingly the significant increase of *Psychrobacter* relative abundance after C&D is only due to the increase in relative abundance of one dominant OTU (cluster 4 in Fig. S1).

**Figure 7:**
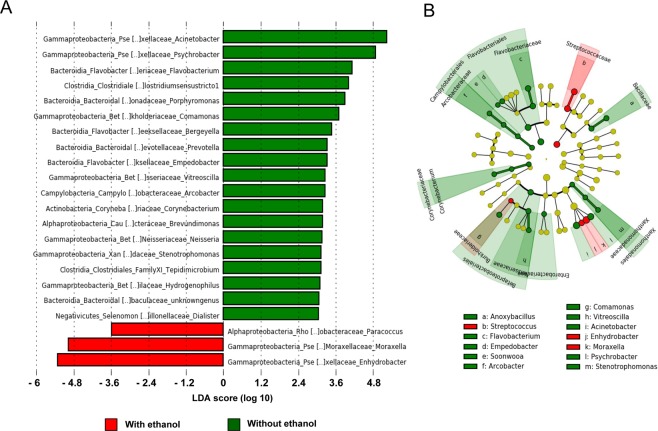
Bacterial genera significantly differentiated between bacterial populations sampled on sampling areas additionally treated with ethanol solution (NC and CO areas) identified by linear discriminant analysis coupled with effect size (LefSE). (**A**) LDA score obtained for differentially abundant taxa. Only genera with a significant LDA threshold value of >3 are displayed. (**B**) Circular cladogram reporting LEfSe results presenting the identified taxa distributed according to phylogenetic characteristics.

The impact of the ethanol-based disinfection on cutting blades at NC and CO areas was also investigated using LEfSE tool. As presented on Fig. 7, *Paracoccus*, *Moraxella* and *Enhydrobacter* genera were statistically more abundant at these sampling areas compared to the others where no additional ethanol-based disinfection step was applied. Conversely, various genera including especially *Psychrobacter* and *Acinetobacter* for the dominant ones were less abundant at areas where additional ethanol-based disinfection was performed.

## Discussion

### Prevalence of *Salmonella* and associated serotypes

In the present work, *Salmonella* was identified at various areas in a pig slaughterhouse. Results revealed that there was no significant difference between the number of detections of *Salmonella*-positive samples before and after C&D procedures highlighting the ability of *Salmonella* to persist in the slaughterhouse environment despite C&D procedures applied. However, it should be noted that the *Salmonella* presence was not specifically quantified. Therefore, even if an important reduction of *Salmonella* levels was operated throughout the C&D procedures, it was not observable here as we investigated only the presence or absence of *Salmonella* after an enrichment step. Serotypes identified are common for the pig and pork sector and in agreement with previous observations^34,35^. We observed in particular a high prevalence of the monophasic variant of Typhimurium serotype exhibiting resistance to various antibiotics (ampicillin, tetracycline and sulfamethoxazole) which accounted for 58% of total *Salmonella* isolates. Such observations are consistent with its fast emergence reported since a decade and its increasing role in human infections^36,37^ and highlight the importance to pay specific attention to this serotype. Some PFGE-patterns were recurrently isolated at various locations in the slaughterhouse and for different dates. In one hand, our results could reflect a persistent colonization of the slaughterhouse, especially as some recurrent strains (such as B01 or B12) were also found before and after C&D for some sampling areas at a given date, underlining their ability to persist despite hygienic procedures. The potential cross-contamination of incoming pig carcasses during the slaughtering process throughout contact with *Salmonella*-positive surfaces in the slaughterhouse may occur at some sites and therefore represent a risk for food safety^5,34,38,39^. On the other hand, it could not be excluded that some strains are also highly prevalent in pigs and were thus continuously introduced in the slaughterhouse with new pig bands to be slaughtered. Strains with PFGE-patterns B04, B09 and B10 have been especially very frequently isolated in French pig slaughterhouses from meat, carcass or feces (unpublished internal data).

The fact that *Salmonella* was not isolated from NC area could be related to the location of this area, just after a singeing step that likely enabled the removal of *Salmonella* from carcasses and thus limited the contamination of the NC area. In addition, the fact that NC was also disinfected using ethanol-based solution should participate to maintain a low level of *Salmonella* contamination at this area. The carcass opening at the next step (CO) and the potentially associated intestinal perforation could then enable *Salmonella* to transfer from intestinal tract to CO surfaces, explaining why *Salmonella* strains were isolated at CO and then at following sampling areas. An increase of *Salmonella* prevalence on pig carcasses was indeed mostly observed after carcass opening and evisceration step^40,41^. Together, these observations again pointed out the criticality of the carcass opening step in controlling *Salmonella* risk at slaughterhouses as already mentioned^5,42^.

### Microbial community diversity and link with *Salmonell*a ecology

The sequencing and analysis of the v3-v4 region of the rDNA 16S sequences revealed that bacterial communities in the slaughterhouse were clearly dominated by the *Moraxellaceae* family and especially four genera: *Acinetobacter*, *Moraxella*, *Psychrobacter* and *Enhydrobacter*. From our knowledge, it is the first 16s rDNA analysis of microbial diversity from pig slaughterhouse environment, therefore there is no available data for direct comparison in the literature. Nevertheless, various published analyses of pig gut microbiome showed the dominance of Firmicutes, Bacteroidetes and Proteobacteria phyla in agreement with the data reported here^43–46^. Such observations are indeed coherent with the fact that bacterial populations found on the slaughterhouse surfaces are initially originating from pig carcasses and then colonize the slaughterhouse environment. In line with this observation, by evaluating the nasal microbiota composition in slaughter-aged pigs using 16s rDNA sequencing, Weese *et al*.^47^ found that *Moraxella*, *Psychrobacter* and *Acinetobacter* were among the most dominant genera in healthy pigs with relative abundance of 35.4, 21.1 and 4.8% respectively, in consistency with data presented here.

The site-specific composition and the stability of populations observed here over the different visits at most of the sampling areas suggest a persistent colonization of the slaughterhouse by the four dominant genera from *Moraxellaceae* family. These observations were supported by the analysis of composition of these genera at the OTUs level (Fig. S1) showing that only 1 or 2 persistent OTUs are found along the slaughter line and for the different sampling dates for the *Psychrobacter*, *Enhydrobacter* or *Moraxella* genera. Survival of bacteria in industries is mostly related to their ability to form biofilms on surfaces exhibiting high tolerance to biocidal treatments^48^. Therefore, it should be interesting to analyze biofilm formation and associated biocide resistance of these resident strains to better understand the mechanisms underlying their persistence in the slaughterhouse.

Analyzing the correlation between the presence of *Salmonella* and abundance of other bacterial taxa in surface populations, we found that some genera were significantly differentially abundant between salmonella-positive or -negative populations. Concerning dominant genera identified in the slaughterhouse, there was a clear negative correlation between *Salmonella* and *Enhydrobacter* genus. Indeed, the dominance of *Enhydrobacter* genus at the NC area was correlated with an absence of *Salmonella* for all sampling dates and interestingly *Enhydrobacter* genus being represented mainly by one single OTU in the slaughterhouse (Fig. S1). Therefore, it could be noteworthy to isolate this strain and test the existence of specific interactions with *Salmonella* using dedicated interaction assays to attribute the absence of *Salmonella* to a specific antagonistic interaction with the *Enhydrobacter* strain or only as a consequence of the singeing step applied before NC area. Reciprocally, positive correlation was found between *Acinetobacter* genus and *Salmonella* presence that may suggest positive interactions between both genera. Overall, such observations should be confirmed by repeating the analysis via a second sampling campaign, giving a view of microbial ecology in the slaughterhouse over the years.

### Impact of C&D procedure on *Salmonella* AMR and bacterial ecology

In one hand, comparison of MIC values obtained for *Salmonella* strains before and after C&D procedures did not reveal significant changes in susceptibilities of isolates against the 14 antibiotics tested. These results suggested here that C&D procedures performed in the slaughterhouse did not lead to the selection of *Salmonella* isolates with increase resistance toward antimicrobials during the time of the study (from March to May 2017). Gantzhorn *et al*.^49^ found similar results while reporting no difference in MICs toward antibiotics nor in the number of resistances of *Salmonella* isolates obtained before and after C&D in six Danish pig slaughterhouses. However, by analyzing susceptibilities of *Pseudomonas* spp. isolated from goat and lamb slaughterhouses to biocides and antibiotics, Lavilla-Lerma *et al*.^50^ found statistical correlations between both antimicrobials revealing a co- or cross-resistance between antimicrobials especially between polyhexamethylene guanidine hydrochloride or triclosan and different antibiotics. First, the relatively short observation time of two months in the present study may not enable to observe an evolution of *Salmonella* antimicrobials susceptibilities. It could thus be interesting to go back in the same slaughterhouse and to sample the same areas to compare the antimicrobial resistance profiles of *Salmonella* strain on a longer period. Furthermore, it would be interesting to also collect data about antimicrobial resistance phenotypes of the associated dominant and persistent genera identified here in the slaughterhouse (especially *Acinetobacter*, *Moraxella*, *Psychrobacter* and *Enhydrobacter*). Indeed, these resident populations undergo repeatedly C&D procedures and are more likely to adapt in the long term. Even though they are not necessarily pathogenic strains, they can represent a potential risk throughout interactions with pathogens as they could play a role in pathogen persistence and/or constitute a potential reservoir for antimicrobial resistances for example.

On the other hand, C&D procedures applied here modified bacterial community composition and led to a loss of richness and evenness in bacterial populations as revealed by the significant decrease of all α-diversity indexes. Likewise, different studies highlighted a loss of diversity in microbial populations after exposure to various disinfectants and active substances including quaternary ammonium-based solutions^51,52^, chlorine^53^, dibromonitrilopropionamide^54^, and other molecules and mixed disinfectants^55^. Analyzing the impact of the additional ethanol-based disinfection on cutting blades, we also found that some genera as *Moraxella*, *Enhydrobacter and Paracoccus* were significantly more abundant at sampling areas treated with ethanol suggesting a potential role of ethanol disinfection step in shaping bacterial populations at NC and CO areas.

The comparison of unifrac and weighted unifrac muldimensional scaling and PERMANOVA analyses (Fig. 4E,F) showed that C&D procedures impact was overall less observable when clustering samples by integrating abundance data and not only presence/absence of OTUs. This observation illustrates that C&D rather led to an elimination of minority OTUs while it had a lower impact on population composition with regards to dominant ones. In addition, LEfSe analysis revealed that while an important number of bacterial orders indeed displayed a significant decrease of their relative abundance after C&D, few genera were favored including *Bergeyella*, *Brevundimonas*, and especially *Rothia* and *Psychrobacter*. Interestingly, *Psychrobacter* was also identified as one of the dominant genus in the slaughterhouse. Furthermore, one single dominant OTU (cluster 4 in Fig. S1) is responsible for the increase in relative abundance observed at all the sampling areas for *Psychrobacter* genus after C&D. From this observation, this strain could therefore constitute a relevant model for identifying markers of bacterial adaptation in such food environments.

## Conclusion

In this contribution, original data on *Salmonella* prevalence and associated microbial ecology in a pig slaughterhouse were collected. The high prevalence of the monophasic variant of Typhimurium and the dominance of the *Moraxellaceae* family along the slaughtering chain were highlighted. Additional 16s rDNA analyses of microbial ecology should be performed over years in this slaughterhouse and also extend to a larger number of pig slaughterhouses in order to confirm such results. Complementary experiments should also be conducted to assess the existence of potential specific interactions between *Salmonella* and resident bacteria identified here (as inhibition with *Enhydrobacter* or positive interactions with *Acinetobacter* for instance). Interestingly, C&D procedures did not lead to the selection of *Salmonella* with higher resistance toward antibiotics and biocides but markedly impacted the composition of bacterial populations associated to slaughterhouse surfaces. From these observations, it seems essential to study antimicrobial resistance dynamics in industries by integrating the whole bacterial community using global approach as shotgun metagenomics. Indeed, resident flora (as *Psychrobacter* here) constitutes the best illustration of bacterial adaptation in a given area and could participate to the dissemination of antimicrobial resistance throughout interactions with pathogens. Overall, data collected here are important for the construction of a realistic vision of *Salmonella* ecology and AMR emergence in food industries with a focus on the potential role of C&D procedures.

## Supplementary information

Supplementary material
