# Supplementary material for: Impact of cleaning and disinfection procedures on microbial ecology and Salmonella antimicrobial resistance in a pig slaughterhouse
Source: Sci Rep. 2019 Sep 10;9:12947. doi: 10.1038/s41598-019-49464-8 (PMC6736965; doi:10.1038/s41598-019-49464-8)
Supplement: Supplementary file 1 — Supplementary material [file 41598_2019_49464_MOESM1_ESM.docx]

*Supplementary material*

**Impact of cleaning and disinfection procedures on microbial ecology and *Salmonella* antimicrobial resistance in a pig slaughterhouse**

Arnaud Bridier ^1,2^, Patricia Le Grandois ^1^, Marie-Hélène Moreau ^1^, Charleyne Prénom^3^, Alain Le Roux ^4^, Carole Feurer ^4^, Christophe Soumet ^1,2^

^1^ Antibiotics, Biocides, Residues and Resistance Unit, Fougères Laboratory, ANSES, Fougères, France; ^2^ Chlean Pass Joint Technological Network: Hygienic Design of Production Lines and Equipment, France;^3^ Department of Fresh and Processed Meat, IFIP-Institut du Porc, Maisons-Alfort, France; ^4^ Department of Fresh and Processed Meat, IFIP-Institut du Porc, Le Rheu, France

**Figure S1. Mean relative abundance of the different OTUs (clusters) in the four dominant genera before (A) and after (B) C&D per sampling area.** DH: dehairing, WH: whipping, NC: neck-clipper, CO: carcass opener, WOG: white offal gutter, ROP: red offal platform.


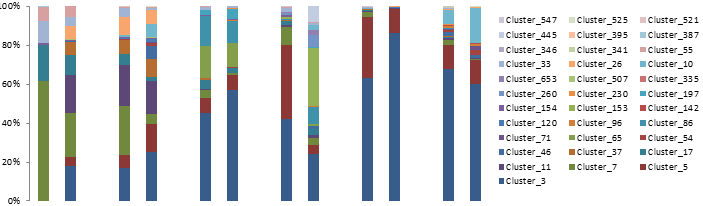

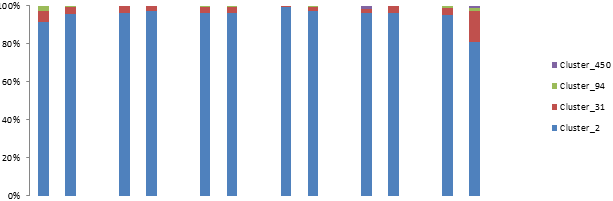

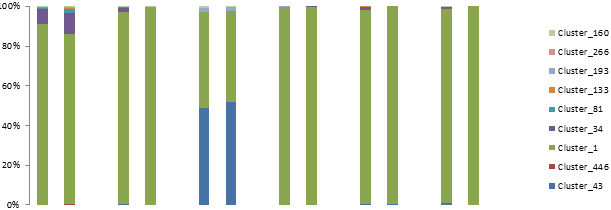

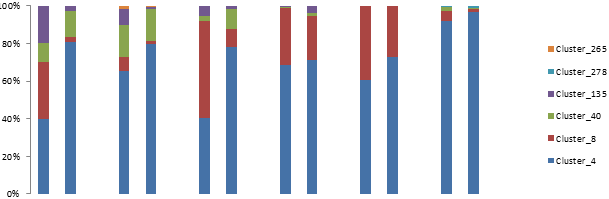


A

B

***Acinetobacter***

***Enhydrobacter***

***Moraxella***

***Psychrobacter***

A

B

A

B

A

B

A

B

A

B

**DH**

**WH**

**NC**

**CO**

**WOG**

**ROP**
